# The Use of Stunkard’s Figure Rating Scale to Identify Underweight and Overweight in Chinese Adolescents

**DOI:** 10.1371/journal.pone.0050017

**Published:** 2012-11-26

**Authors:** Wing-Sze Lo, Sai-Yin Ho, Kwok-Kei Mak, Tai-Hing Lam

**Affiliations:** School of Public Health, University of Hong Kong, Pokfulam, Hong Kong; Virginia Commenwealth University, United States of America

## Abstract

**Background:**

To compare the performance of Stunkard’s current body size (CBS) with self-reported body mass index (BMI), waist circumference (WC) and waist to stature ratio (WSR) in predicting weight status in Chinese adolescents, and to determine the CBS cutoffs for overweight/obesity and underweight.

**Methodology:**

This cross-sectional study was conducted in a sample of 5,418 secondary school students (45.2% boys; mean age 14.7 years). Height and weight were measured by trained teachers or researchers. Subjects were classified as underweight, normal weight, or overweight/obese according to the International Obesity Task Force cutoffs. Subjects were asked to select the figure that best resembled their CBS on the Stunkard’s figure rating scale. Self-reported height, weight, WC and WSR were also obtained. The performance of CBS, self-reported BMI, WC and WSR as a weight status indicator was analysed by sex-specific receiver operating characteristic curves. The optimal CBS cutoffs for underweight and overweight/obesity were determined based on the Youden Index.

**Principal Findings:**

Apart from self-reported BMI, CBS had the greatest area under curve (AUC) for underweight in boys (0.82) and girls (0.81). For overweight/obesity, CBS also had a greater AUC (0.85) than self-reported WC and WSR in boys, and an AUC (0.81) comparable to self-reported WC and WSR in girls. In general, CBS values of 3 and 5 appeared to be the optimal cutoffs for underweight and overweight/obesity, respectively, in different sex-age subgroups.

**Conclusions/Significance:**

CBS is a potentially useful indicator to assess weight status of adolescents when measured and self-reported BMI are not available.

## Introduction

Adolescent weight status is commonly defined using body mass index (BMI), which is weight (kg) divided by height squared (m^2^). When direct measurements of height and weight are infeasible, self-reports are often used. However, over-reporting of height and under-reporting of weight are common [1], [2], [3], [4], and adolescents might have difficulty reporting them. Waist circumference (WC) and waist to stature ratio (WSR) are emerging indicators of central obesity and cardiovascular risk [5], [6], [7], [8]. These indicators, involving measurements of WC and height, can also be self-reported by adolescents, although the use of such data in predicting weight status was seldom reported.

In contrast, figure rating scale is a simple visual tool to assess body image and perceived body size [9]. The commonly used Stunkard’s figure rating scale [2] comprises a series of nine male or female figure drawings of increasing body size. Figural stimuli have been used in psychological research to assess ideal body size and current body size (CBS) in adults [2], [10], [11]. CBS has also been used in epidemiological studies to assess weight status in adults [2], [9], [11]. It accurately delineated underweight and overweight in receiver operating characteristic (ROC) analysis, with area under curve (AUC) over 0.85 in men and women [11]. However, little is known whether CBS could be used to predict weight status in adolescents, and which figures best delineate underweight and overweight from normal weight [1]. Without involving any numerical estimation of height, weight or WC, CBS is relatively easy to administer and comprehend [12], especially to adolescents whose rapid growth may render self-reported anthropometric measures out-dated and inaccurate [1]. CBS may also have implications for use in clinical and educational settings.

We have reported that the validity and test-retest reliability of CBS are acceptable in Chinese adolescents [13]. In the present study, we compared CBS with self-reported BMI, WC and WSR as indicators of weight status in Chinese adolescents, and determined CBS cutoffs for underweight and overweight/obesity.

## Materials and Methods

### Ethics Statement

Ethical approval was granted by the Institutional Review Board of the University of Hong Kong/Hospital Authority Hong Kong West Cluster.

### Study Population and Study Design

The present study is part of a large school-based survey, the Hong Kong Student Obesity Surveillance (HKSOS) project. Details of the sampling method have been reported elsewhere [14], [15]. Briefly, a stratified cluster sample of 42 schools was recruited, representing all mainstream non-international secondary schools in Hong Kong by district, source of funding, language of instruction (Chinese/English), religious background (Christian/others/none) and single sex/co-education. All secondary 1 to 7 (equivalent to grade 7 to 12 in the United States) students in the selected schools were invited to voluntarily complete an anonymous questionnaire in Chinese about obesity with height and weight self-reported (n = 22612). An invitation letter with a reply slip for refusal was sent to the parents, whose consent for participation was assumed unless the signed reply slip was returned.

Among the participating schools, 15 measured anthropometric data of students (n = 6753). After excluding 778 students with incomplete anthropometric data, 309 with extreme self-reported BMI values (<10 kg/m^2^ or >50 kg/m^2^) [16], [17] and 248 aged over 18, 5418 (2451 boys and 2967 girls; aged 12–17; mean age 14.7 [1.6] years) remained for analysis. To evaluate the reliability of the questionnaire, 788 students from two girl schools and one co-education school (29.2% boys, mean age 14.9 [1.7] years) completed a retest after four weeks.

### Measures

Height (cm) and weight (kg) were measured barefoot and in light clothing by trained teachers or researchers, following an established protocol [18]. Height, weight and waist circumference (WC, cm) were also self-reported by participants to the nearest integer. BMI was calculated as weight (kg) divided by height squared (m^2^). The self-reported BMI of the subjects included in the analysis was comparable with that of the whole sample (Cohen effect size d = 0.04) [19]. Waist to stature ratio (WSR) was calculated as WC divided by height.

Using the Stunkard’s figure rating scale, the students selected from 9 male or female body figures of increasing size (labelled 1–9) that best resembled their CBS [2].

### Analyses

Based on measured height and weight, weight status was classified as underweight, normal weight, overweight or obese according to the International Obesity Task Force age- and sex-specific BMI cutoffs [20], [21]. These cutoffs for underweight, overweight and obesity correspond to adult BMI values of 17, 25 and 30, respectively [20], [21]. Due to their small number, obese subjects were combined with the overweight as overweight/obese. ROC curves were generated based on the measured weight status (reference method), and were then used to calculate the AUC of CBS and self-reported BMI, WC and WSR for underweight and overweight/obesity in each sex. An AUC of 0.5 (or 50%) indicates performance of no better than chance, while an AUC of 1.0 (or 100%) indicates perfect discrimination of cases (underweight or overweight/obesity) from non-cases. The Youden index (J), calculated using the formula J = sensitivity+specificity −1 [22], is a derivative of the sum of sensitivity and specificity ranging from 0 to 1. The CBS cutoff value for a weight status (e.g. underweight) that gave the greatest Youden index denoted the optimal cutoff for that weight status, as it corresponds to the point on the ROC curve farthest from chance [23]. Apart from Youden index, positive likelihood ratios (LR+; LR+ = sensitivity/1-specificity) were also calculated. LR+ summarises the performance of diagnostic tests by taking into account both sensitivity and specificity [23]. The larger the LR+, the more likely a positive test result predicts the presence of the condition, which is underweight or overweight/obesity in the present study. LR+ of 5–10, 2–5 and <2 indicate good, fair, and poor performance, respectively [24]. CBS differences between weight status categories were analyzed by one-way ANCOVA adjusting for age. All analyses were conducted using SPSS version 17.0 [25].

## Results

Based on measured data, boys were significantly taller (p<0.001), heavier (p<0.001) and had greater BMI (p<0.001) than girls. The overall prevalence of underweight, overweight and obesity in the total sample was 5.7%, 9.9% and 1.2%, respectively (Table 1). The four-week test-retest reliability Spearman correlations (*r*) for CBS were 0.72 for boys and 0.78 for girls, and the correlations between measured and self-reported BMI were 0.75 for boys and 0.79 for girls (both p<0.001). Underweight boys had a mean CBS of 2.81 (95% confidence interval [CI] 2.60, 3.01), whereas normal weight and overweight/obese boys had a mean CBS of 4.03 (3.98, 4.08) and 5.65 (5.54, 5.76), respectively. The mean CBS in girls was 2.48 (2.36, 2.61) for underweight, 3.59 (3.55, 3.62) for normal weight and 4.84 (4.72, 4.97) for overweight/obese. CBS differed by weight status in both boys (p<0.001) and girls (p<0.001).

**Table 1:** Measured and self-reported anthropometric characteristics in boys and girls.

		Boys(n = 2451)	Girls(n = 2967)
Age (years, mean, SD)		14.7 (1.6)	14.8 (1.6)
Measured data	Height (cm, mean, SD)	165.0 (9.1)	157.6 (5.8)
	Weight (kg, mean, SD)	53.7 (11.3)	47.7 (7.6)
	BMI (mean, SD)^a^	19.6 (3.3)	19.2 (2.7)
Weight status (n, %)^b^	Underweight	110 (4.5)	200 (6.7)
	Normal weight	1941 (79.2)	2563 (86.4)
	Overweight/obesity	400 (16.3)	204 (6.9)
	Overweight	343 (14.0)	194 (6.5)
	Obesity	57 (2.3)	10 (0.3)
Self-reported data	Height (cm, mean, SD)	165.4 (9.4)	157.7 (5.8)
	Weight (kg, mean, SD)	53.3 (11.2)	47.0 (7.5)
	BMI (mean, SD)^a^	19.4 (3.3)	18.9 (2.7)
	WC (cm, mean, SD)^c^	69.4 (18.0)	64.6 (17.5)
	WSR (mean, SD)^d^	0.42 (0.1)	0.41 (0.1)
CBS (mean, SD)^e^		4.2 (1.3)	3.6 (1.0)

a BMI = body mass index.

b weight status defined based on IOTF references and measured height and weight.

c WC = waist circumference;

d WSR = waist to stature ratio;

e CBS = current body size.

**Figure 1:**
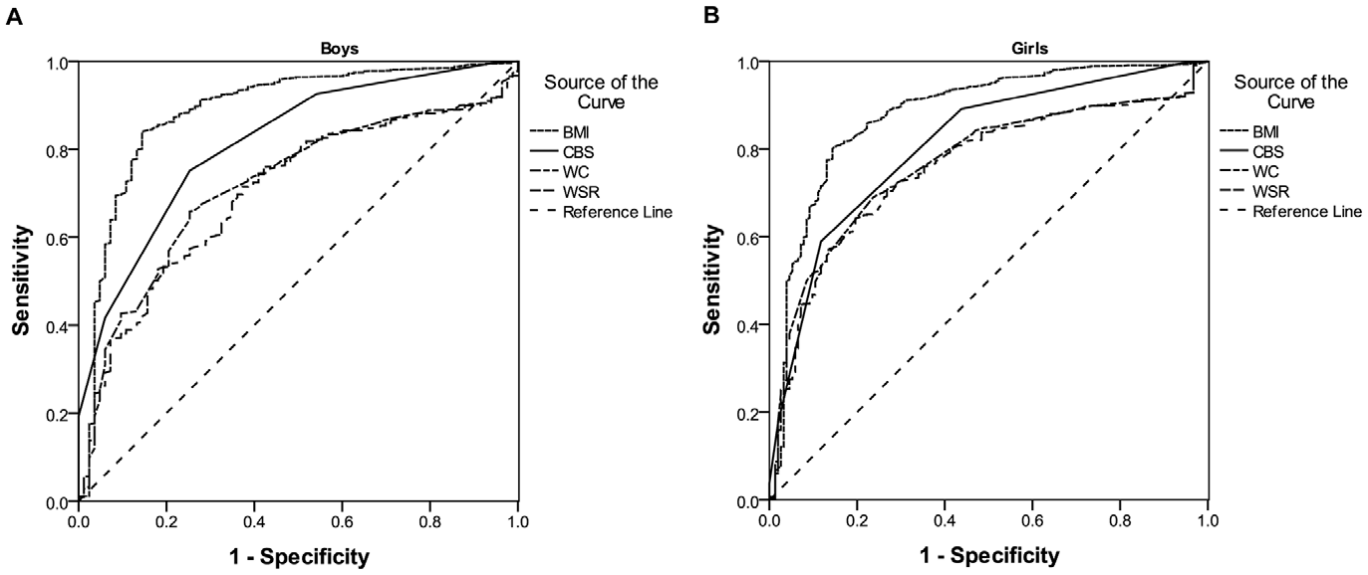
Receiver operating characteristic (ROC) curves for underweight in boys and girls. Apart from BMI, CBS had the greatest AUC in both boys and girls. (A) AUC for boys: self-reported BMI (0.89); CBS (0.82); self-reported WC (0.72); self-reported WSR (0.70). (B) AUC for girls: self-reported BMI (0.88); CBS (0.81); self-reported WC (0.77); self-reported WSR (0.76).

**Figure 2:**
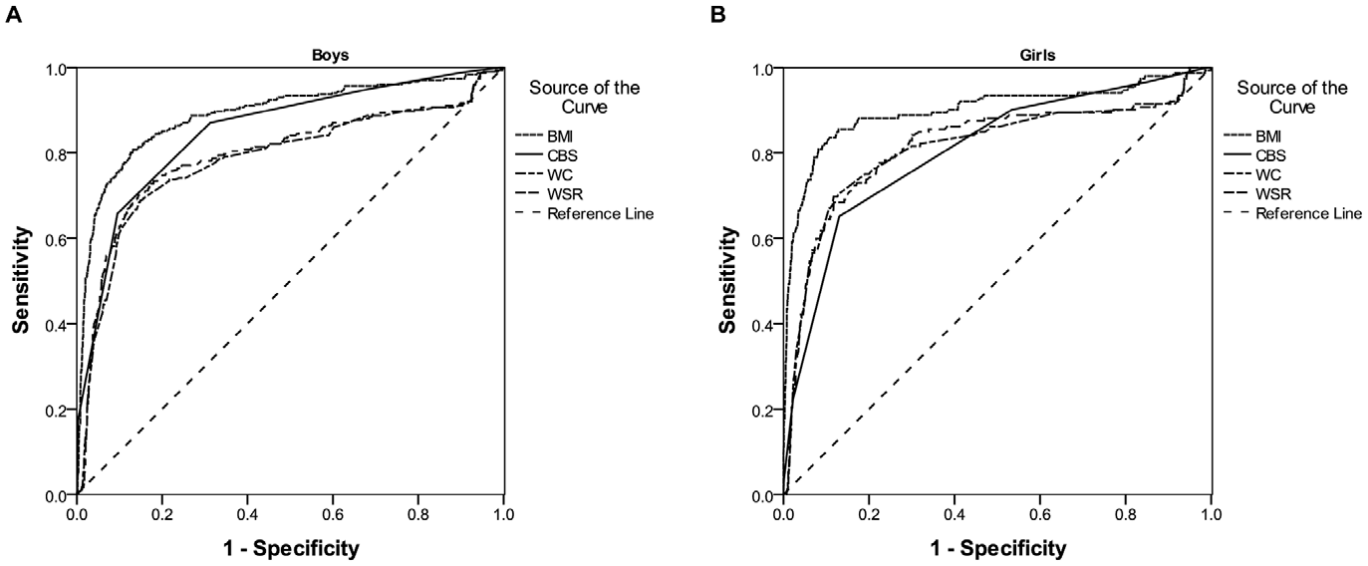
Receiver operating characteristic (ROC) curves for overweight/obesity in boys and girls. Self-reported BMI had the greatest AUC for both sexes. In boys, CBS had a higher AUC than WSR and WC, while in girls the AUCs of CBS, WSR and WC were comparable. (A) AUC for boys: self-reported BMI (0.89); CBS (0.85); self-reported WC (0.80); self-reported WSR (0.78). (B) AUC for girls: self-reported BMI (0.90); CBS (0.81); self-reported WC (0.82); self-reported WSR (0.82).

Apart from BMI (0.89 for boys and 0.88 for girls), CBS had the greatest AUC in both boys (0.82) and girls (0.81) for underweight (Figure 1). The corresponding AUCs for overweight/obesity were shown in Figure 2. Self-reported BMI had the greatest AUC for both sexes (0.89 for boys and 0.90 for girls). In boys, CBS (0.85) had a higher AUC than WSR (0.80) and WC (0.78), while in girls the AUCs of CBS (0.81), WSR (0.82) and WC (0.82) were comparable.

Based on the Youden index, CBS values of 3 and 5 appeared to be the optimal cutoffs in general for underweight and overweight/obesity, respectively, in different sex-age subgroups (Table 2). In general, higher sensitivity was observed in boys and higher specificity was observed in girls. The LR+ was fair to good for the cutoffs. Using the CBS cutoffs, the prevalence of underweight and overweight/obesity was 37.3% and 27.1%, respectively.

**Table 2:** Optimal current body size cut-offs for overweight/obesity and underweight by sex and age.

Age (years)			Underweight					Overweight/obesity				
		n (%)	CBS	Sensitivity	Specificity	J	LR+	CBS	Sensitivity	Specificity	J	LR+
Boys	All	2451	3	0.748	0.764	0.512	3.2	5	0.870	0.690	0.560	2.8
	12–<13	359 (14.6)	3	0.696	0.882	0.578	5.9	5	0.788	0.734	0.522	3.0
	13–<14	522 (21.3)	3	0.697	0.792	0.488	3.3	5	0.856	0.742	0.597	3.3
	14–<15	527 (21.5)	3	0.779	0.850	0.629	5.2	5	0.900	0.689	0.589	2.9
	15–<16	468 (19.1)	3	0.759	0.737	0.496	2.9	6	0.762	0.889	0.651	6.9
	16–<17	338 (13.8)	3	0.811	0.500	0.311	1.6	5	0.973	0.671	0.644	3.0
	17–<18	237 (9.7)	3	0.758	0.900	0.658	7.6	6	0.619	0.898	0.517	6.1
Girls	All	2967	3	0.580	0.885	0.465	5.0	5	0.652	0.873	0.525	5.1
	12–<13	455 (15.3)	2	0.830	0.645	0.475	2.3	5	0.500	0.954	0.454	11.0
	13–<14	577 (19.4)	3	0.538	0.935	0.474	8.3	5	0.672	0.894	0.566	6.3
	14–<15	581 (19.6)	3	0.901	0.611	0.512	2.3	5	0.654	0.873	0.527	5.2
	15–<16	571 (19.2)	3	0.625	0.814	0.439	3.4	5	0.774	0.850	0.624	5.2
	16–<17	413 (13.9)	3	0.643	0.906	0.549	6.9	5	0.650	0.842	0.492	4.1
	17–<18	370 (12.5)	3	0.658	0.889	0.547	5.9	5	0.800	0.816	0.616	4.4

CBS: current body size; J: Youden index; LR+: positive likelihood ratio.

## Discussion

CBS, self-reported BMI, WC and WSR all predicted weight status reasonably well with AUCs over 0.70. As expected, self-reported BMI had the greatest AUC because a BMI-derived weight status standard was adopted. As such, self-reported BMI was more of a reference for the greatest achievable AUC. Among the other measures, CBS was apparently the best, having the greatest AUC for underweight in boys and girls. It also had a greater AUC than self-reported WC and WSR for overweight/obesity in boys, and an AUC comparable to self-reported WC and WSR in girls. Due to the media hype around female body shape, girls may be more susceptible to body image distortions, which may partly explain their lower AUC for overweight/obesity than boys. Previous studies have shown that weight misperceptions were more common in girls than boys [26], which is in line with our current observations. To our knowledge, CBS has not been used to assess weight status in adolescents but several adult studies exist [2], [9], [11]. The accuracy/AUCs of CBS for the identification of underweight and obesity have been high in both men (underweight & obesity: 0.88) and women (underweight: 0.87; obesity: 0.93) in the United States [11], although direct comparisons are difficult due to differences in age, ethnicity and weight status standard used.

Moreover, compared with self-reported WC and WSR, CBS has the advantage of visualising adiposity of the whole body. Adolescents may not know their WC well as it is seldom measured in schools or at home [27], especially among boys [28]. Also, WC may be measured at different sites in adolescents and no international agreement on the optimal site is available [29], [30], [31]. WC reference values for adolescents, developed only recently [7], have yet to be widely used to assess weight status.

In girls, these cutoffs were more specific than they were sensitive in detecting overweight/obesity or underweight, which again suggested overestimation of CBS in some girls. LR+ is often used in the clinical setting to evaluate the usefulness of diagnostic tests [32]; the higher the LR+, the more likely an individual with a positive test result has the weight problem. The LR+ of our CBS cutoffs was generally fair to good in different sex-age subgroups. No obvious trend was observed for the variations in sensitivity, specificity, J and LR+ by age. The variations could be due to the relatively small sample size (200–500 students) in each subgroup. Nevertheless, the CBS cutoffs were generally stable (3 for underweight and 5 for overweight/obesity) in most subgroups, except for underweight girls aged 12 (cutoff of 2), and for overweight/obese boys aged 15 and 17 (cutoff of 6), but the differences were small.

In general, our CBS cutoffs are similar to those of previous research [1], [11], despite the differences in age, ethnicity, body composition and the use of weight status references. Using the Stunkard’s figure rating scale [2], figure number 3 for underweight and figure number 7 for obesity have been suggested for American men and women, but the cutoff for overweight was not determined [11]. On the other hand, based on expert opinion from 108 clinicians and researchers, Must et al. [1] suggested that figure number 4 was at 50^th^ BMI percentile (defined by the CDC BMI-for-age growth charts [33]), and figure numbers 5 and 6 corresponded to the cutoffs of overweight (85^th^ percentile) and obesity (95^th^ percentile), respectively, in adolescent girls.

The prevalence rates of underweight and overweight/obesity based on the CBS cutoffs were markedly higher than those based on the International Obesity Task Force standard, suggesting that CBS is sensitive in detecting underweight and overweight/obesity for screening purposes. Screened positive adolescents should then be examined objectively to determine weight status. Early intervention could be implemented when needed, thus preventing long-term psychosocial and health consequences.

Although the performance of CBS was lower than self-reported BMI, CBS can be particularly useful when height and weight are not well recalled, especially in young children and adolescents who may have difficulty reporting their anthropometric data or when participants are reluctant to reveal [1]. It can also be used to estimate the body size of peers, siblings and family members, which is an important attribute of the social environment to adolescent obesity [34]. Physicians and teachers can use CBS as a quick screening tool to assess and record the weight status of adolescents if objective measurements were infeasible, especially in places like Hong Kong where physicians were lack of consultation time, space and appropriate equipment for routine anthropometric measurement checkup [35]. Moreover, only one CBS cutoff was proposed for each of underweight and overweight/obesity regardless of age and sex. In contrast, the varying International Obesity Task Force cutoffs by sex and age for adolescents [21] make BMI difficult to use in community settings.

The strengths of the present study are the large, representative sample and the inclusion of four different anthropometric indicators for comparison. However, the use of measured BMI as a surrogate measure for general adiposity is a limitation [36], although it is the most feasible and well-established assessment for body fatness in large epidemiologic studies [20], [37]. Percentage body fat measured using leg-to-leg bioimpedance is a potential alternative but well-accepted cutoffs are lacking. Another problem is that we could not estimate the cutoffs for overweight and obesity separately due to the small number of obese subjects. Moreover, although 42 schools were originally included in the study, only 15 schools had objectively measured height and weight. It is a routine to collect height and weight annually on all secondary students during physical education classes in Hong Kong, but it is not compulsory for teachers to record waist circumference or save anthropometric data systemically for later use. The small sample size in specific age groups (age 12 and age 17) might also have affected the precision of estimates. Finally, as the CBS cutoffs identified were based on Chinese adolescents, they may not be applicable to other adolescent populations due to potential differences in body size and body compositions.

### Conclusions

The third and the fifth Stunkard’s figure drawings were identified as the CBS cutoffs for underweight and overweight/obesity, respectively, in Chinese adolescent boys and girls aged 12–17. CBS is a potentially useful indicator to assess weight status of adolescents when measured and self-reported BMI are not available. The use of CBS to assess the weight status of oneself and others warrants further investigations in different ethnic and age groups.
